# The APT complex is involved in non-coding RNA transcription and is distinct from CPF

**DOI:** 10.1093/nar/gky845

**Published:** 2018-09-21

**Authors:** Michael Lidschreiber, Ashley D Easter, Sofia Battaglia, Juan B Rodríguez-Molina, Ana Casañal, Manuel Carminati, Carlo Baejen, Pawel Grzechnik, Kerstin C Maier, Patrick Cramer, Lori A Passmore

**Affiliations:** 1 Department of Molecular Biology, Max Planck Institute for Biophysical Chemistry, Göttingen, Germany; 2 Karolinska Institutet, Department of Biosciences and Nutrition, Center for Innovative Medicine and Science for Life Laboratory, Novum, Hälsovägen 7, 141 83 Huddinge, Sweden; 3 MRC Laboratory of Molecular Biology, Cambridge CB2 0QH, UK; 4 School of Biosciences, University of Birmingham, Edgbaston, Birmingham B15 2TT, UK

## Abstract

The 3′-ends of eukaryotic pre-mRNAs are processed in the nucleus by a large multiprotein complex, the cleavage and polyadenylation factor (CPF). CPF cleaves RNA, adds a poly(A) tail and signals transcription termination. CPF harbors four enzymatic activities essential for these processes, but how these are coordinated remains poorly understood. Several subunits of CPF, including two protein phosphatases, are also found in the related ‘associated with Pta1′ (APT) complex, but the relationship between CPF and APT is unclear. Here, we show that the APT complex is physically distinct from CPF. The 21 kDa Syc1 protein is associated only with APT, and not with CPF, and is therefore the defining subunit of APT. Using ChIP-seq, PAR-CLIP and RNA-seq, we show that Syc1/APT has distinct, but possibly overlapping, functions from those of CPF. Syc1/APT plays a more important role in sn/snoRNA production whereas CPF processes the 3′-ends of protein-coding pre-mRNAs. These results define distinct protein machineries for synthesis of mature eukaryotic protein-coding and non-coding RNAs.

## INTRODUCTION

RNA polymerase II (Pol II) synthesizes protein-coding mRNAs as well as a number of non-coding RNAs including snoRNAs, snRNAs, lncRNAs and miRNAs. Pol II requires additional proteins for transcription including initiation, elongation and termination factors, as well as proteins involved in processing of the nascent RNA transcript. One of these is the Cleavage and Polyadenylation Factor (CPF in yeast, or CPSF in higher eukaryotes), which carries out 3′-end processing of mRNAs (1,2).

CPF is an ∼1 MDa complex that cleaves pre-mRNAs with an endonuclease activity found within the Ysh1 subunit (CPSF73 in higher eukaryotes). The polymerase subunit Pap1 (PAP in higher eukaryotes) adds adenosines to the newly generated 3′-end in a template-independent manner. In addition, two subunits contain protein phosphatase activity: Ssu72 can dephosphorylate Ser5 and Ser7 of the C-terminal domain (CTD) of the largest subunit of Pol II during transcription elongation, whereas Glc7 dephosphorylates CTD Tyr1 and facilitates transcription termination (3). The enzymatic activities of CPF—endonuclease, polymerase and phosphatase—must be tightly coupled to transcription and to each other to ensure that the pre-mRNA is cleaved only once, the poly(A) tail is synthesized to an appropriate length before mRNA is exported from the nucleus, and transcription termination occurs in a timely manner.

**Figure 1.**
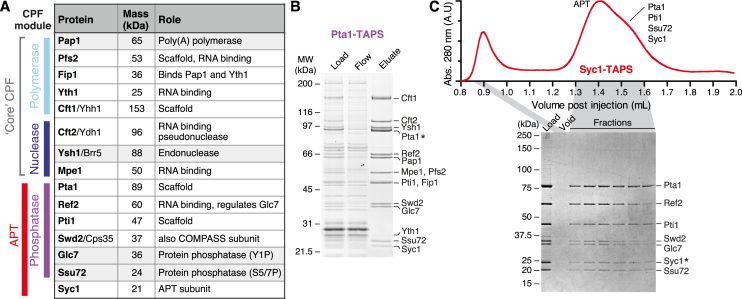
Purification of CPF and APT complexes. (**A**) CPF subunits from yeast. Names of proteins used in this work are in bold. Masses were calculated using *ProtParam*. (**B**) SDS-PAGE analysis of Pta1-TAPS purification from Streptactin resin, stained with SYPRO Ruby. Subunits were analyzed using tryptic-digest mass spectrometry from excised bands and all 15 previously-known CPF proteins were identified. (**C**) Purification from a Syc1-tagged yeast strain yields only the seven APT subunits. The gel filtration chromatogram (absorbance at 280 nm, arbitrary units) and corresponding Coomassie-blue stained SDS-PAGE analysis of fractions are shown. An asterisk indicates the tagged subunit.

Tagging of CPF subunits in yeast has allowed purification of intact endogenous CPF complex that contains the endonuclease, polymerase and phosphatase activities (4–8). However, the exact composition of the complex and assembly of the subunits had remained elusive. Early work showed that the phosphatase subunits were part of the seven subunit APT (associated with Pta1) complex (6). It was suggested that APT dynamically associates with a smaller ‘core CPF’ complex containing Cft1, Cft2, Ysh1, Pta1, Pap1, Mpe1, Pfs2, Yth1 and Fip1 to form a 15-subunit ‘holo-CPF’. More recently, we used native mass spectrometry to show that the CPF complex is assembled via three stably-associated modules based around the three enzymatic activities (9). Thus, CPF is comprised of nuclease, polymerase and phosphatase modules (Figure 1A). Still, because native mass spectrometry only provided insight into the composition of CPF subcomplexes, and not the entire CPF, it remained unclear how the three modules assemble to form CPF.

Mutation of either the RNA sequences that specify the polyadenylation site or the 3′-processing machinery results in transcriptional readthrough on protein-coding genes (8,10–12). In addition, reverse transcription and northern blot experiments show an involvement of APT subunits and the accessory Cleavage Factor (CF) IA subunits in preventing transcriptional readthrough from some (but not all) snoRNAs, and chromatin immunoprecipitation (ChIP) on individual genes revealed that APT subunits cross-link to snoRNAs and the 3′-ends of mRNA genes (6,7,13–21). Both of the CPF phosphatases are implicated in transcription termination of snoRNAs. In contrast, there is no strong evidence linking other CPF subunits with snoRNA transcription and, although snoRNAs are transcribed by Pol II, their mature forms are not polyadenylated. SnoRNA transcription termination is generally independent of Ysh1 endonuclease cleavage and is instead dependent on the Nrd1/Nab3/Sen1 (NNS) pathway (18,22–24).

Syc1 (similar to Ysh1 C-terminus) is the smallest protein to be identified as a subunit of APT/CPF (4,6,25) but it was not consistently identified in all preparations. Here, we use chromatographic methods to show that Syc1 is the defining subunit of a distinct APT complex, and is not part of CPF. The other six APT subunits are, however, also present in CPF, forming the phosphatase module. The phosphatase module/APT subunits play a separable role on non-coding RNAs, revealing a global function in transcription of a subclass of Pol II transcripts. These results suggest that synthesis of mature coding and non-coding Pol II transcripts primarily involves CPF and APT complexes, respectively.

## MATERIALS AND METHODS

### Yeast strains

*Saccharomyces cerevisiae* (*Sc*) strains containing genes with C-terminal TAPS-tags for protein purification were constructed as previously described (8,9). For ChIP and PAR-CLIP, *Sc* BY4741 strains containing C-terminally TAP-tagged genes (Open Biosystems) were tested for expression of the correctly tagged protein. For 4tU-seq and ChIP-qPCR, knockout strains in *Sc* BY4741 (Open Biosystems), or JWY104 were generated as previously described (26,27) and verified via PCR. For inducible snR47 experiments, strains were generated by a one-step PCR procedure (28,29) using the *GAL1* (lacking *GAL1* UTR) promoter amplified by PCR from the pFA6a-His3Mx6-pGAL1 plasmid with oligos 5GLSNR47 and 3GLSNR47 2mce (Supplementary Table S1).

### Purification of CPF

CPF was isolated using affinity chromatography as previously described (8), with the following modifications. For the purifications in Supplementary Figures S1B and S2, the 48 litre yeast cultures were harvested and resuspended in lysis buffer (200 mM HEPES pH 8.0, 200 mM KCl, 0.5 mM Mg(OAc)_2_ and 10% w/v glycerol). The cells were frozen and lysed by a Freezer Mill 6870 (SPEX CertiPrep). The crude lysate was then centrifuged at 45,000 rpm in a Ti45 rotor for 1 h before loading onto IgG Sepharose (8).

The complex was further purified/analyzed using size exclusion chromatography (SEC) or anion exchange. SEC was performed as follows: CPF was concentrated using Amicon Ultra 0.5 ml 50 kDa cut-off centrifuge concentrators (Millipore), clarified at 16,100 *g*, and injected onto a 2.4 ml Superose 6 PC 3.2/30 column (GE) pre-equilibrated in 20 mM HEPES pH 7.9, 150 mM KCl, 3 mM DTT, 0.5 mM Mg(OAc)_2_, 0.5 mM MnCl_2_. 50 μl fractions were collected. In a second independent experiment, CPF and APT complexes were separated using anion exchange: The sample isolated from the affinity step (8) was injected into a 1 ml Mono Q 5/50 GL column (GE Healthcare) equilibrated in 20 mM HEPES pH 7.9, 150 mM KCl, 3 mM DTT, 0.5 mM Mg(OAc)_2_, and eluted in a two-step or linear gradient. APT eluted at 260 mM KCl and CPF eluted at 400 mM KCl.

### Analysis of native CPF purifications

SDS-PAGE analysis was performed using Novex 4–12% Bis–Tris gels with NuPAGE MOPS SDS running buffer (Life Technologies). Gels were stained with Coomassie Blue or SYPRO Ruby (Lonza), as indicated in figure legends. On SDS-PAGE, Cft1 bands were observed to run as smears if the gel got too hot; this was overcome by limiting the maximum current and ensuring buffer covered the entire gel. Mpe1 and Pfs2 ran very close to one another, as did Pti1 and Fip1. Fip1 was identified as the band immediately below Pti1 (although other studies suggested the opposite migration pattern, (4,30)). All CPF subunits were identified using tryptic-digest mass spectrometry via LC-MS/MS on a Velos Orbitrap ESI spectrometer (Thermo) from excised gel bands. Spectra were analyzed against Mascot databases (Matrix Science) and filtered using *Scaffold* software (Proteome Software) to show the relevant *S. cerevisiae* hits.

### Functional genomics

ChIP-seq and PAR-CLIP were carried out as described (31). 4tU-seq was carried out as described (32). ChIP-seq and 4tU-seq experiments were carried out as biological replicates. ChIP-qPCR of Pta1-TAPS and Cft2-TAP was carried out as described previously (33) with minor modifications. Briefly, Pta1-TAPS was immunoprecipitated from WT or Δ*syc1* cells using Streptactin sepharose resin and eluted with 2 mg/ml desthiobiotin in elution buffer (50 mM Tris–HCl pH 7.4, 10 mM EDTA, 1% SDS) at room temperature for 30 min. Cft2-TAP was eluted with TEV protease in 20 mM HEPES pH 7.9, 150 mM KCl, 0.5 mM MgCl_2_, 0.5 mM Mg(OAc)_2_, 3 mM DTT at 4°C overnight. Oligonucleotide sequences used for qPCR analysis are detailed in Supplementary Table S1.

### Northern blot analysis of inducible snR47

Strains were grown at 30°C in SC medium (0.67% yeast nitrogen base, supplemented with the required amount of amino acids and nucleotide bases) containing 2% raffinose and 0.08% glucose to OD_600_ = 0.5. Transcription from the *GAL1* promoter was induced by addition of 2% galactose. Total RNA was isolated using a hot phenol procedure (34). For Northern blot analysis, 2 μg of total RNA was separated on either 6% polyacrylamide-urea TBE or 1% agarose-formaldehyde MOPS gel. RNA was transferred onto nylon membrane (GE Healthcare) by electrotransfer (for polyacrylamide gel; in TransBlot Biorad, 100 mA for 60 min) or capillary transfer (for agarose gels). RNA was visualized on the blot by methylene blue staining (0.3 M sodium acetate pH 5.3, 0.02% methylene blue). Hybridization with oligonucleotides Snr47so2 (snR47), S13so (snR13) or Gal1PCRsonR (GAL1) (Supplementary Table S1) labelled with ^32^P at their 5′-ends was performed for 5–12 h at 42°C in PerfectHyb buffer (Sigma) and followed by three washes with 6× SSPE. Hybridization signals were visualized using BioRad imaging system.

### Bioinformatics analysis

#### Transcript annotation and filtering

We used TIF-Seq data from (35) to derive TSS and pA site annotations for 5578 protein-coding genes. TSS and 3′-end positions were taken from the *Saccharomyces* Genome Database (SGD, version = R64.2.1) for snRNAs and snoRNAs (referred to as sn/snoRNAs) and from (32) for CUTs. Unless stated otherwise, for PAR-CLIP (ChIP-seq) analyses, annotated mRNA and sn/snoRNA transcripts were filtered to be at least 150 nt (200 bp) away from neighboring transcripts on the same (both) strand(s). Since we were interested in comparing mRNA and sn/snoRNA transcript classes, we further filtered our gene sets so that overlapping mRNA and sn/snoRNA loci (e.g. sn/snoRNAs located within introns of mRNAs) were excluded from all analyses. Filtering reduced the number of analyzed sn/snoRNAs from 83 to 62 (PAR-CLIP) and 29 (ChIP-seq) and mRNAs from 5578 to 2967 (PAR-CLIP) and 724 (ChIP-seq).

#### ChIP-seq data analysis

ChIP-seq data analysis was performed as described (31) with some modifications. Briefly, paired-end 50 bp reads were aligned to the *S. cerevisiae* genome (sacCer3, version 64.2.1) using the short read aligner *Bowtie* (version 2.2.3) (36). *SAMTools* was used to quality filter SAM files (37). Alignments with MAPQ smaller than 7 (-q 7) were skipped and only proper pairs (-f99, -f147, -f83, -f 163) were selected. Further processing of the ChIP-Seq data was carried out using the *R/Bioconductor* environment. Piled-up counts for every genomic position were averaged over replicates, using physical coverage, that is, counting both sequenced bases covered by reads and unsequenced bases spanned between proper mate-pair reads. Normalization between IP and Input was done using the signal extraction scaling (SES) factor obtained with the estimateScaleFactor function from *deepTools* (38) with options: –l 100 –n 100 000 and the median fragment size (-f) estimated from the data (∼200 bp). ChIP enrichments were obtained by dividing SES-normalized IP intensities by the corresponding input intensities: log2(IP/Input). Whereas relative ChIP-Seq signals can be readily compared for one factor between different genes, comparison of ChIP-Seq signals from different factors at the same genes is challenging. Thus, to better quantify differences in occupancies, we converted the log2(IP/Input) signals for each factor to occupancies by setting the genome-wide 99.8% and 10% log_2_(IP/Input) quantiles to 100% and 0% occupancy, respectively (39). Resulting normalized occupancy profiles were smoothed (sliding window averaging, window half size of 50 bp) before further analysis.

#### PAR-CLIP data analysis

PAR-CLIP data analysis was performed as described (31) with some modifications. Briefly, quality-trimmed reads were aligned to the *S. cerevisiae* genome (sacCer3, version 64.2.1) using the short read aligner *STAR* (version 2.5.2b; options: –outFilterMultimapNmax 1, –outFilterMismatchNmax 1, –scoreDelOpen −10 000, –scoreInsOpen −10000, –alignSJoverhangMin 10000, –alignSJstitchMismatchNmax 0 0 0 0 (40)). The resulting SAM files were then converted into BAM and PileUp files using *SAMTools* (37).

We calculated the *P*-values for true crosslinking sites as described (41). Briefly, we had to quantitatively model the null hypothesis, that is, the probability that the T-to-C mismatches observed in reads covering a certain T nucleotide in the genome were not caused by cross-links between the immunoprecipitated factor and RNA but are due to the other sources of mismatches. Owing to the exquisite sensitivity of our experimental PAR-CLIP procedure, we could set a very stringent *P*-value cut-off of 0.005 and a minimum coverage threshold of two. For true crosslinking sites passing our stringent thresholds, the PAR-CLIP-induced T-to-C transitions strongly dominate over the contributions by sequencing errors and SNPs. For any given T site in the transcriptome, the number of reads showing the T-to-C transition is proportional to the occupancy of the factor on the RNA times the concentration of RNAs covering the T site. Therefore, the occupancy of the factor on the RNA is proportional to the number of reads showing the T-to-C transition divided by the concentration of RNAs covering the T site. This concentration was estimated by the read coverage obtained from a RNA polymerase (Pol) II (Rpb1 subunit) PAR-CLIP experiment (31) and was used to obtain normalized occupancies. Pol II (Rpb1) normalized occupancy profiles were smoothed (sliding window averaging, window half size of 50 nt) and used for further analysis. To compare averaged, normalized RNA-binding occupancies between transcript classes, they were scaled together by setting min (transcript class 1, transcript class 2) to 0 and max (transcript class 1, transcript class 2) to 1. Motif analysis using *XXmotif* (42) did not reveal any enriched motifs around the strongest Syc1 binding sites.

#### 4tU-Seq data analysis

Data analysis was performed as described (32), with minor modifications. Briefly, paired-end 50 bp reads with additional 6 bp of barcodes were obtained for labelled RNA on an Illumina 2500 sequencer. Reads were demultiplexed and aligned to the *S. cerevisiae* genome (sacCer3, version 64.2.1) using *STAR* (version 2.3.0) (43). *SAMTools* was used to quality filter SAM files (37). Alignments with MAPQ smaller than 7 (−q 7) were skipped and only proper pairs (−f99, −f147, −f83, −f163) were selected. Further processing of the RNA-Seq data was carried out using the *R/Bioconductor* environment. Piled-up counts for every genomic position were averaged over replicates, using physical coverage, that is, counting both sequenced bases covered by reads and unsequenced bases spanned between proper mate-pair reads. Fold changes of newly synthesized RNA levels between Δ*syc1* and wild-type cells were calculated using the *R/Bioconductor* implementation of *DESeq2* (44) setting betaPrior = FALSE. Per gene read counts were calculated after mapping using *HTSeq* (45) for all mRNAs and sn/snoRNAs except loci where mRNAs and sn/snoRNAs overlapped (see above). Before applying *DESeq2*, the count matrix was further filtered to contain only genes with at least 30 averaged, normalized read counts in either wild-type or knockout condition resulting in 4801 and 43 candidate mRNA and sn/snoRNA genes, respectively. Differentially expressed genes were identified applying a fold change cutoff of 1.5 and an adjusted *P*-value cutoff of 0.1. *DESeq* size factors were not only used for differential expression analysis but also to normalize piled-up, gene-averaged coverage profiles.

## RESULTS

### CPF and APT are distinct endogenous complexes

We purified the CPF complex by tagging the Pta1 subunit with a tandem-affinity purification ‘TAPS’ tag, comprised of a double Strep-II tag separated by a TEV protease site from a C-terminal protein A moiety tag (8). Tagged CPF was purified on IgG sepharose, eluted with TEV protease, bound to Streptactin resin and finally eluted with desthiobiotin. We chose Pta1 for tagging since previous studies had indicated that it might act as a central scaffold (6). All fifteen known CPF subunits were co-purified, confirmed by mass spectrometry, and there were no substantial contaminants visible on the gel (Figure 1B).

In contrast, when we tagged the smallest subunit, Syc1, the only associated proteins were the six proteins previously identified as APT subunits, namely Pta1, Ref2, Pti1, Swd2, Glc7 and Ssu72 (Figure 1C). The intact APT complex migrated as a peak on a gel filtration column with a second trailing peak consisting of a subcomplex of Pta1, Pti1, Ssu72 and Syc1. These experiments confirmed the subunit composition of CPF and suggested a distinct subunit composition of the APT complex that we investigated further.

### Syc1 is a subunit of APT but not CPF

Purification of tagged Syc1 suggested that it wasn’t tightly associated with CPF. However, it had remained unclear from our work and other studies, whether the APT complex exists separately from CPF or whether tagging of Syc1 disrupts its association with CPF. Therefore, to gain insight into the organization of CPF/APT and the relationships between their subunits, we systematically tagged different subunits and analyzed the purified components. We then examined association of Syc1 with CPF complexes using size exclusion chromatography.

**Figure 2.**
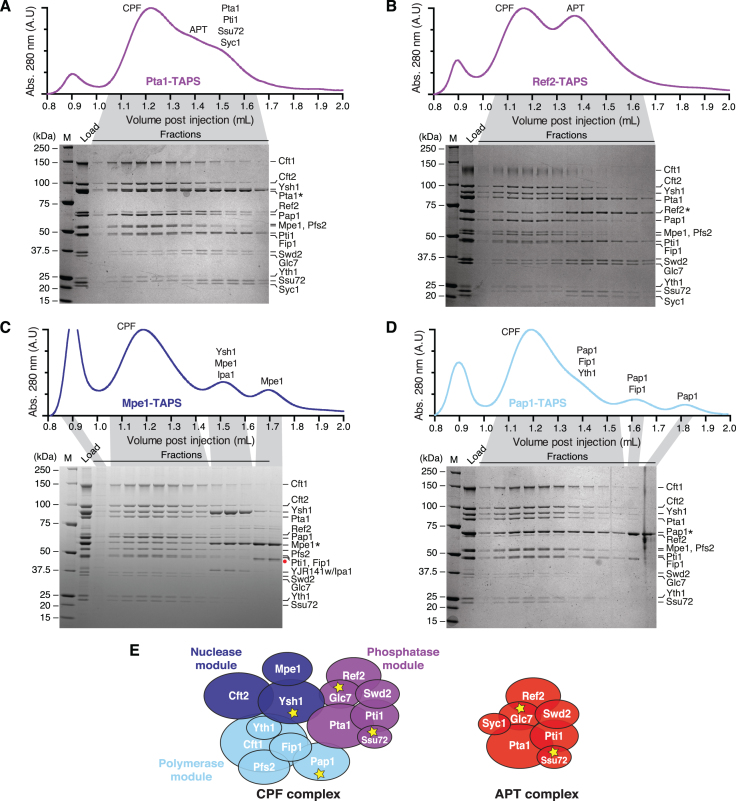
Syc1 is not found in the CPF complex. (A–D) Purifications from (**A**) Pta1-, (**B**) Ref2-, (**C**) Mpe1- and (**D**) Pap1-tagged yeast strains. Gel filtration chromatograms (absorbance at 280 nm, arbitrary units) and corresponding Coomassie-blue stained SDS-PAGE analyses of fractions are shown. Asterisks indicate the tagged subunits. The red circle (panel C) represents a degradation product of Mpe1 identified by tryptic digest mass spectrometry. M is molecular weight marker. (**E**) Schematic diagrams of CPF and APT complexes. Proteins have an area proportionate to their molecular weight. Yellow stars denote enzymes.

Purifications from Pta1- and Ref2-tagged strains (phosphatase module subunits) each contained two distinct complexes that could be separated using size exclusion chromatography: a 14-subunit CPF complex with all subunits except Syc1, and a 7-subunit APT complex (Figure 2A, B). Purifications from Ref2-tagged strains yielded the most homogeneous complexes, where the overall stoichiometry of most subunits within CPF and APT complexes appeared to be close to uniform. This may be because Ref2 is a limiting component.

Phosphatase module/APT subunits did not dissociate from the CPF complex after consecutive runs (and dilution) on a gel filtration column, suggesting that it is a stable and separate complex (Supplementary Figure S1A). Moreover, both APT and CPF could be isolated from a TAPS preparation from Ref2-tagged yeast using anion exchange chromatography (Supplementary Figure S1B).

In contrast, purifications from strains with tagged Mpe1 (nuclease module) or Pap1 (polymerase module) did not contain any Syc1 (Figure 2C, D). Thus, a tag on a polymerase or nuclease module subunit, which we define as the ‘core’ CPF, only purifies the 14 CPF subunits, whereas a tagged phosphatase module subunit also co-purifies Syc1 and the APT complex, and tagged Syc1 only purifies APT (Supplementary Figure S1C). These data suggest that Syc1 is the defining subunit of APT, which exists as a separate complex from CPF in the cell. We refer to the Syc1-bound complex as APT, whereas CPF-bound Pta1-Pti1-Ref2-Ssu72-Swd2-Glc7 is the phosphatase module.

To further assess whether APT formation is dependent on Syc1, we purified proteins from a Pta1-tagged strain where *SYC1* had been deleted. We were able to isolate CPF, but not APT or isolated phosphatase module, on anion exchange chromatography from this strain (Supplementary Figure S2). Therefore, APT formation/stability is dependent on Syc1. Notably, we were unable to obtain a yeast strain where Ref2 was C-terminally tagged and *SYC1* was deleted, indicative of a synthetic lethal interaction.

Examination of the gel filtration profiles also revealed the presence of subcomplexes whose composition agrees with our previous model of CPF architecture generated from nanoelectrospray ionization mass spectrometry (nanoESI-MS) data (9): Pta1-TAPS preparations included the Pta1-Pti1-Ssu72-Syc1 subcomplex (Figure 2A). In Mpe1-tagged purifications, a subcomplex of Ysh1-Mpe1-Ipa1/Yjr141w could be separated from CPF on gel filtration columns (Figure 2C). Ipa1 was recently shown to genetically interact with CPF subunits (46) but the function of the Ysh1-Mpe1-Ipa1 complex remains unclear. Purifications from Pap1-tagged yeast contained excess Pap1-Fip1-Yth1 complex, Pap1-Fip1 and isolated Pap1 (Figure 2D).

Taken together, these results revealed that APT and CPF are distinct endogenous complexes in yeast that both contain the six subunits of the phosphatase module. They can be distinguished by the presence and absence, respectively, of the APT-specific subunit Syc1 (Figure 2E).

### CPF and APT subunits occupy protein-coding genes but Syc1 is more abundant on sn/snoRNA genes

**Figure 3.**
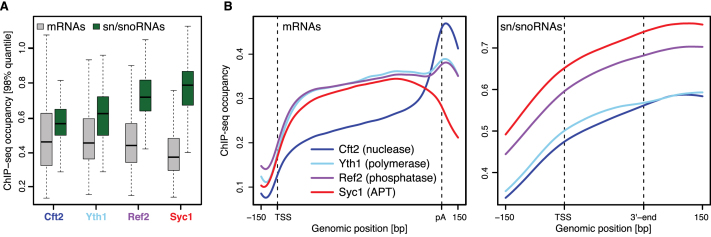
APT is more abundant than CPF on sn/snoRNA genes. (**A**) Distribution of ChIP-seq occupancies at selected mRNA (gray, *n* = 724) and sn/snoRNA (green, *n* = 29) genes (see *Materials and Methods*). Gene-wise ChIP-seq occupancies were derived by taking the 98% quantile of the smoothed, normalized occupancies over the region covering the gene body and 100 bp downstream. Box limits are the first and third quartiles, the band inside the box is the median. The ends of the whiskers extend the box by 1.5 times the interquartile range. (**B**) Gene-averaged ChIP-seq occupancy profiles over selected mRNA (left) and sn/snoRNA (right) genes (as in A). Before averaging, normalized gene profiles were aligned at their transcription start site (TSS) and length-scaled such that their polyadenylation (pA) sites/3′-ends coincided.

We hypothesized that separate APT and CPF complexes could play roles on different gene classes. To investigate whether the APT and CPF complexes differ in their cellular functions, we first used ChIP-seq profiling to quantitate their association with chromatin. We used yeast strains with tagged Cft2 (CPF nuclease module), Yth1 (CPF polymerase module), Ref2 (CPF phosphatase module & APT complex) or Syc1 (APT complex) and measured occupancy of the genome with these subunits by ChIP-seq. Interestingly, CPF and APT ChIP to all types of Pol II-transcribed genes but the occupancies of these proteins were different on mRNA versus sn/snoRNA genes (Figure 3A). Specifically, the APT defining subunit Syc1 was much more abundant (∼2-fold) on sn/snoRNA genes than on mRNA genes.

Metagene profiles of the ChIP data across mRNA genes revealed a peak at the 3′-end for CPF-specific subunits Cft2 and Yth1, whereas Syc1 was depleted (Figure 3B). Concordantly Ref2, which is part of both APT and CPF complexes, was highly abundant on sn/snoRNA genes, similar to Syc1, and also peaked at the 3′-end of mRNA genes, similar to Cft2 and Yth1. The profiles for core CPF subunits Cft2 and Yth1 differ: The reasons for this are unclear but it could be a result of differences in immunoprecipitation efficiency or the dynamic nature of CPF during transcription elongation. CPF and APT were present across gene bodies, in agreement with previous data showing that CPF is recruited early in transcription in yeast and metazoans (6,12,47–49). ChIP-seq occupancies at individual genomic loci agree well with the metagene profiles (Supplementary Figure S3, top panel).

ChIP-qPCR on candidate genes showed that the phosphatase module/APT subunit Pta1 is more abundant on all sn/snoRNA genes tested than on the *PDC1* protein-coding gene (Supplementary Figure S4A). In contrast, Cft2 is more abundant at the 3′-end of *PDC1* than on any of the sn/snoRNAs genes. Taken together, these data show that CPF is more abundant at the 3′-end of mRNA genes, while APT is more abundant on sn/snoRNA genes.

### Syc1 preferentially crosslinks to sn/snoRNA transcripts

**Figure 4.**
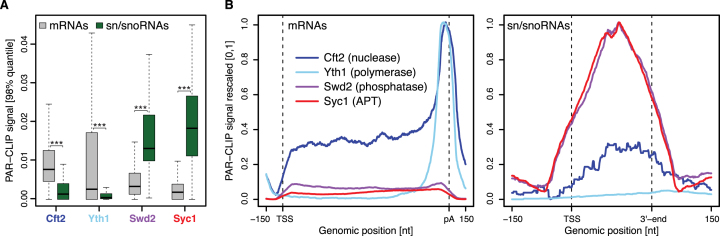
Syc1 preferentially crosslinks to sn/snoRNA transcripts. (**A**) Distribution of PAR-CLIP occupancies at selected mRNAs (grey, *n* = 2905) and sn/snoRNAs (green, *n* = 62) (see *Materials and Methods*). Gene-wise PAR-CLIP occupancies were derived by taking the 98% quantile of the smoothed, normalized occupancies over the region covering the gene body and 100 bp downstream. *P*-values were derived by two-sided Mann–Whitney U test, ***P < 0.001. Box limits are the first and third quartiles, the band inside the box is the median. The ends of the whiskers extend the box by 1.5 times the interquartile range. (**B**) Gene-averaged PAR-CLIP occupancy profiles over selected mRNAs (left) and sn/snoRNAs (right) (as in A). Before averaging, normalized gene profiles were aligned at their TSS and length-scaled such that their pA sites/3′-ends coincided.

Our ChIP-seq data suggested that CPF and APT may play distinct roles on different gene classes. To investigate this further, we performed PAR-CLIP of Swd2 (phosphatase module) and Syc1, and compared these to published PAR-CLIP data of the nuclease and polymerase module subunits Cft2 and Yth1 (41). PAR-CLIP maps RNA-binding proteins on the transcriptome with the use of UV crosslinking (50). This revealed a highly significant difference in mRNA versus sn/snoRNA crosslinking. Cft2 and Yth1 both crosslinked preferentially to mRNA whereas Swd2 and Syc1 were ∼4- and 10-fold more abundant on sn/snoRNAs compared to mRNAs (Figure 4A).

Metagene profiles revealed a striking peak at the 3′-end of mRNAs for Cft2 and Yth1 (Figure 4B). This is expected because this is the site of mRNA cleavage and polyadenylation. In comparison, Swd2 and Syc1 showed low occupancy on mRNAs but strong crosslinking over the body of sn/snoRNA transcripts. The binding pattern in metagene profiles was also visible on individual transcripts (Supplementary Figure S3, middle panel). These results show that APT subunits preferentially crosslink to sn/snoRNA transcripts, consistent with a role of APT in non-coding RNA transcription.

### Deletion of *SYC1* results in decreased sn/snoRNA transcription

Whereas both ChIP and PAR-CLIP occupancy profiles suggested that Syc1 is preferentially associated with sn/snoRNA genes and transcripts, these methods cannot reveal whether Syc1 functions in sn/snoRNA transcription. We therefore investigated RNA synthesis *in vivo* using metabolic labeling with 4-thiouracil, followed by sequencing of the newly synthesized, labeled RNA (4tU-seq) (51,52).

**Figure 5.**
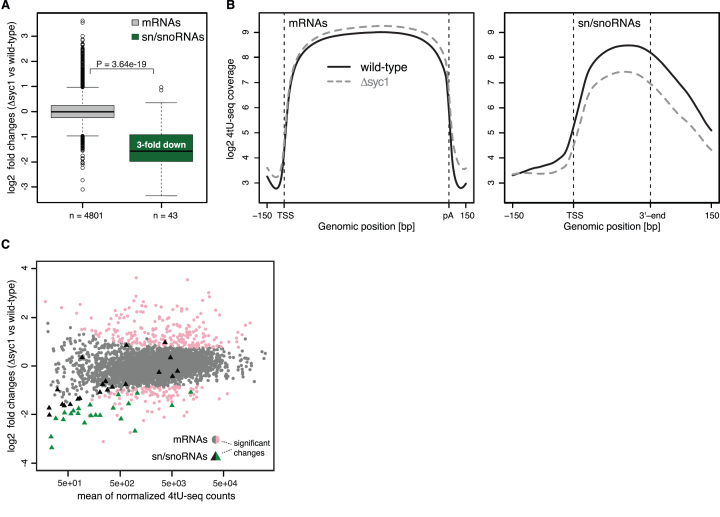
*SYC1* deletion leads to down-regulation of sn/snoRNA transcription. (**A**) Distribution of log2 fold changes of normalized 4tU-RNA-seq read counts for Δ*syc1* versus wild-type cells for selected mRNAs (grey, *n* = 4801) and sn/snoRNAs (green, *n* = 43) (see *Materials and Methods*). The *P*-value was derived by two-sided Mann–Whitney U test. Box limits are the first and third quartiles, the band inside the box is the median. The ends of the whiskers extend the box by 1.5 times the interquartile range. (**B**) Transcript-averaged coverage of newly synthesized RNA measured by 4tU-Seq in wild-type (solid line) and Δ*syc1* (dashed line) yeast over selected mRNAs (left) and sn/snoRNAs (right) (as in A). Before averaging, normalized transcript profiles were aligned at their TSS and length-scaled such that their pA sites/3′-ends coincided. (**C**) MA-plot showing log2 fold change for each transcript between Δ*syc1* and wild-type yeast, versus the normalized mean read count across replicates and conditions. Transcripts with a fold change >1.5 and adjusted *P*-value below 0.1 (as calculated by *DESeq2*, Materials and Methods) are shown in color. mRNAs and sn/snoRNAs are shown as grey/pink circles and black/green triangles, respectively.

Analysis of fold changes between RNA synthesis levels in Δ*syc1* and wild-type strains revealed that sn/snoRNA transcription is globally reduced by a median fold change of ∼3 relative to mRNAs (Figure 5A and Supplementary Figure S3, bottom panel). Metagene profiles of the 4tU-seq data show that reduced transcription in Δ*syc1* yeast occurs across sn/snoRNAs gene bodies (Figure 5B). We also examined transcription of a snoRNA using a yeast strain where the promoter of *SNR47* was replaced with the *GAL1* promoter. After induction with galactose, snR47 accumulated more slowly in the Δ*syc1* strain than in wild-type yeast (Supplementary Figure S4B).

Examination of individual transcripts across the entire 4tU-seq dataset showed that sn/snoRNAs were significantly enriched among the down-regulated genes (*P*-value 1.38e-24, Fisher's exact test). Over 50% of candidate sn/snoRNAs were significantly down-regulated by >1.5-fold (adjusted *P*-value 0.1) (Figure 5C). In contrast, ∼2% of mRNAs (104 in total) were significantly down-regulated and ∼5% (229 in total) were significantly up-regulated by >1.5-fold (adjusted *P*-value 0.1) (Figure 5C). The down-regulated transcripts include those encoding ribosomal proteins, mating response proteins and histones, whereas up-regulated transcripts include those involved in stress response and metabolism of amino acids (Supplementary Figure S5).

ChIP-qPCR on individual genes shows a small but reproducible reduction in the occupancy of Pta1 on some genes that are downregulated in the 4tU-seq dataset in Δ*syc1* compared to wild-type yeast (Supplementary Figure S4A). In contrast, Cft2 occupancy was unchanged when *SYC1* was deleted. Together, these data show that the specific interaction of Syc1/APT with sn/snoRNAs has a functional role in sn/snoRNA transcription *in vivo*. When Syc1 is not present, APT is destabilized (Supplementary Figure S2), resulting in a reduced occupancy of APT on sn/snoRNAs genes (Supplementary Figure S4A) and less efficient transcription (Figure 5).

## DISCUSSION

**Figure 6.**
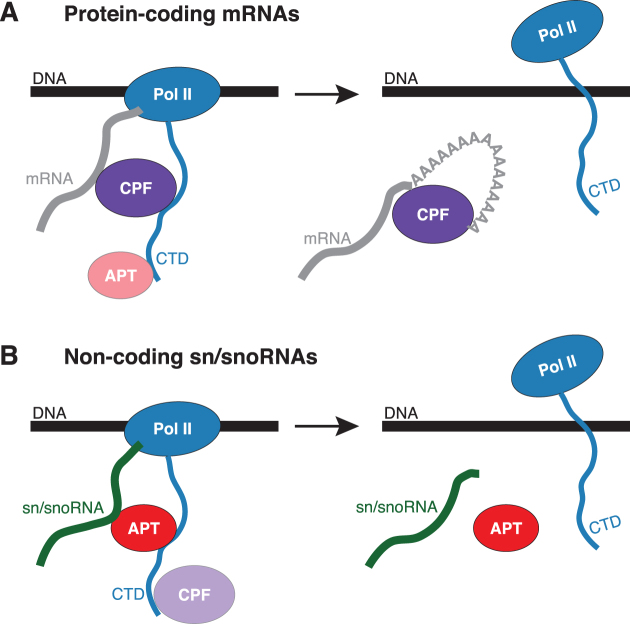
Model for CPF and APT function in transcription. Models are shown for CPF (**A**) and APT (**B**) on protein-coding mRNAs and sn/snoRNAs.

Here we show that yeast contains distinct CPF and APT complexes. APT is defined by the APT-specific subunit Syc1, and additionally contains the six subunits of the phosphatase module of CPF. We further show that Syc1 preferentially occupies non-coding genes and non-coding Pol II transcripts. Finally, we demonstrate that Syc1 is required for normal sn/snoRNA synthesis, whereas its deletion does not largely affect mRNA synthesis *in vivo*. Taken together, these results are consistent with a model where APT plays a more important role in the transcription of non-coding Pol II transcripts, which do not have poly(A) tails, and therefore do not apparently require CPF, which is responsible for 3′-end processing of mRNAs (Figure 6).

### CPF and APT are separate complexes with six overlapping subunits

Syc1 was previously described as a genuine component of CPF because it co-purified with the CPF complex (6). The improved yields and purities of CPF and APT in our preparations reveal that Syc1 only co-purifies with CPF when a phosphatase module subunit is tagged. Tagged nuclease or polymerase module subunits do not co-purify Syc1. Furthermore, the phosphatase module is a stable component of CPF, whereas APT (with Syc1) is not.

In yeast, the C-terminus of Ysh1 can be replaced with the Syc1 sequence, with no loss of function, and it was suggested that the two subunits compete for the same binding site within CPF, thereby regulating 3′-end processing (53). The binding site for Syc1/Ysh1 is likely the C-terminus of Pta1 (6,20). Pta1 does not appear to be super-stoichiometric in CPF and APT complexes, and we did not find two copies of Pta1 in the complex using nano-ESI mass spectrometry experiments (9). Thus, our data are not consistent with two copies of Pta1 in CPF. It is likely that the interactions of Ysh1 and Syc1 with Pta1 are mutually exclusive, and Syc1 in the APT complex occupies the binding site of the C-terminus of Ysh1.

### APT is important for sn/snoRNA transcription

CPF and APT are recruited to both protein-coding and sn/snoRNA gene loci (this study and (18)). Both complexes are recruited to both gene classes so there may be some overlap in their functions. It was previously suggested that recruitment of both CPF and NNS factors to all classes of Pol II transcripts allows elongating Pol II to choose between alternative transcription termination pathways (18). We observe a strong enrichment of Syc1 (APT-specific) over Cft2 and Yth1 (CPF-specific) at sn/snoRNA genes and direct RNA interactions clearly show that Cft2/Yth1 and Syc1 interact specifically with pre-mRNAs and sn/snoRNAs respectively. This suggests a specific role for Syc1/APT in sn/snoRNA biogenesis. We corroborate this finding by showing that sn/snoRNA transcription is down-regulated in a *SYC1* deletion mutant. We were not able to purify APT from a Δ*syc1* strain, and therefore CPF or isolated phosphatase subunits may compensate for loss of full APT. Using PAR-CLIP, we cannot distinguish whether Syc1 binds sn/snoRNAs co- or post-transcriptionally, but the strong Syc1 recruitment to sn/snoRNA gene loci we observe by ChIP suggests that Syc1 engages RNA during transcription.

In the *SYC1* deletion mutant, we also observe down regulation of a small subset of mRNAs, including mRNAs coding for ribosomal proteins. This could be a secondary effect due to reduced sn/snoRNA production. Alternatively, Syc1/APT may coordinately regulate a group of transcripts that are constitutively highly expressed (including ribosomal proteins and histones) by promoting efficient transcription of specific genes. Interestingly, like sn/snoRNAs, yeast histone mRNA production is influenced by Sen1 (54). Since both gene classes are down-regulated on *SYC1* deletion, APT and Sen1 function may be linked, in agreement with previous data (19).

The phosphatase module of CPF regulates Pol II transcription by dephosphorylating CTD Ser5/Ser7 and Tyr1 with its Ssu72 and Glc7 subunits, respectively. This allows co-ordination of transcription with mRNA 3′-end processing. In contrast, APT regulates Pol II transcription of non-coding transcripts (Figure 6). This model is consistent with the finding that Syc1 has no effect on *in vitro* cleavage and polyadenylation (53). While some APT subunits (e.g. Ssu72) may regulate termination of sn/snoRNA transcription, others (Ref2, Pta1, Pti1) do not (6). We did not observe transcriptional readthrough in the *SYC1* deletion mutant. Interestingly, the PAR-CLIP metagene profile for Syc1 is not overlapping with that for Nrd1 (Supplementary Figure S6) (52). Because there is very little overlap between Syc1 and Nrd1 on RNA, their binding may be sequential. Whereas Nrd1 promotes transcription termination through recognition of GUAA/G terminator sequence motifs downstream of the mature 3′-end of sn/snoRNAs (52), we did not find a particular motif enriched around the strongest Syc1 PAR-CLIP sites. This suggests that Syc1/APT might recognize specific sn/snoRNA secondary structures or another feature of these genes/transcripts. One interesting possibility is that CPF and APT could contribute to anti-terminator activity during transcription elongation for canonical termination and NNS-mediated termination respectively. At the 3′-end of the gene, action of the phosphatases could relieve the anti-terminator activity, promoting efficient transcription termination at the correct site (8). Deletion of *SYC1* would not have a strong phenotype since aberrantly terminated transcripts in the absence of Syc1 would be rapidly degraded. Rapid degradation of aberrant transcripts may also explain the decreased transcription of sn/snoRNAs we observe in this strain.

Syc1 is conserved among yeasts but an orthologous protein has not been identified in other eukaryotes. Thus, an APT-dependent mechanism of snoRNA synthesis may have evolved in yeast where most snoRNAs are independent transcription units. In contrast, most metazoan snoRNAs are encoded within the introns of protein-coding genes and their transcription may therefore depend on CPF/CPSF. For snRNAs, the Integrator complex, which is related to CPF, is used for 3′ processing in metazoans (55).

Cloning of a canonical polyadenylation/termination signal at the 3′-end of snoRNAs causes cleavage and polyadenylation, and prevents production of functional, mature product (13). In addition, core CPF subunits are not required for synthesis of mature sn/snoRNAs. Thus, we propose that APT, in combination with the CF IA subunit Pcf11 (18,56,57), is required for production of mature sn/snoRNAs. It is likely that sequence features, Pol II CTD phosphorylation state, and additional protein factors contribute to whether APT- or CPF-mediated gene regulation occurs. Taken together, this work defines APT as a distinct complex and reveals a function of the single subunit that differs from CPF subunits, Syc1, in the transcription of non-coding Pol II transcripts.

## DATA AVAILABILITY

ChIP-seq, PAR-CLIP and 4tU-RNA-seq datasets have been deposited in GEO under accession number GSE114304 (https://www.ncbi.nlm.nih.gov/geo/query/acc.cgi?acc=GSE114304).

## Supplementary Material

Supplementary Data
