# Supplementary material for: The APT complex is involved in non-coding RNA transcription and is distinct from CPF
Source: Nucleic Acids Res. 2018 Sep 21;46(21):11528–38. doi: 10.1093/nar/gky845 (PMC6265451; doi:10.1093/nar/gky845)
Supplement: Supplementary Data [file gky845_supplemental_files.pdf]

SUPPLEMENTARY FIGURES AND LEGENDS

The APT complex is involved in non-coding RNA transcription and is distinct from CPF

Michael Lidschreiber[1,2,†]*, Ashley D. Easter[3,†], Sofia Battaglia[1], Juan B. Rodríguez-Molina[3], Ana Casañal[3], Manuel Carminati[3], Carlo Baejen[1], Pawel Grzechnik[4], Kerstin C. Maier[1], Patrick Cramer[1,2,]* and Lori A. Passmore[3,]*

[1] Department of Molecular Biology, Max Planck Institute for Biophysical Chemistry, Göttingen, Germany
[2] Karolinska Institutet, Department of Biosciences and Nutrition, Center for Innovative Medicine and Science for Life Laboratory, Novum, Hälsovägen 7, 141 83 Huddinge, Sweden
[3] MRC Laboratory of Molecular Biology, Cambridge CB2 0QH, UK
[4] School of Biosciences, University of Birmingham, Edgbaston, Birmingham, B15 2TT, UK

* To whom correspondence should be addressed. Tel: +44 1223 267062; Email: passmore@mrc-lmb.cam.ac.uk
Correspondence may also be addressed to M.L., Email: michael.lidschreiber@ki.se or P.C., Email: pcramer@mpibpc.mpg.de

[†] The authors wish it to be known that, in their opinion, the first 2 authors should be regarded as joint First Authors.

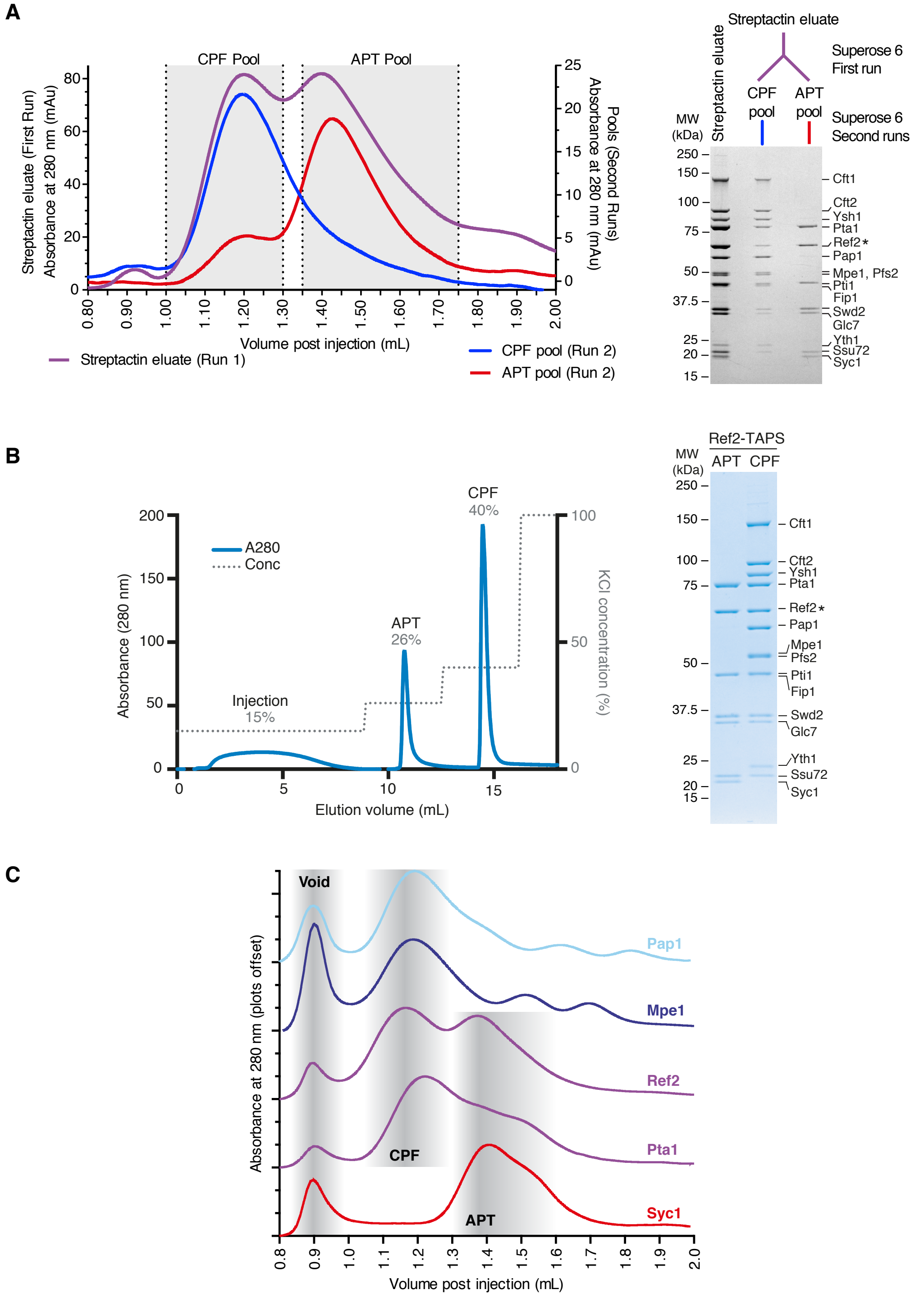

Supplementary Figure S1: Syc1 is in a separate complex from CPF. (**A**) Absorbance traces at 280 nm for consecutive Superose 6 PC 3.2/30 size exclusion chromatography runs (left). Coomassie-stained SDS-PAGE analysis of concentrated Streptactin eluate from the first run, and pooled CPF and APT fractions after second runs on size exclusion chromatography (right). (**B**) Protein purified from a Ref2-TAPS strain was analyzed by anion exchange chromatography, which separated APT and CPF complexes. A representative chromatogram is shown on the left and a representative Coomassie-stained SDS-PAGE gel of the two peak fractions is shown on the right. In (A) and (B), an asterisk indicates the tagged subunit. (**C**) Overlay of chromatograms from analyses of CPF and APT complexes on Superose 6 size exclusion chromatography (see Figures 1 and 2).

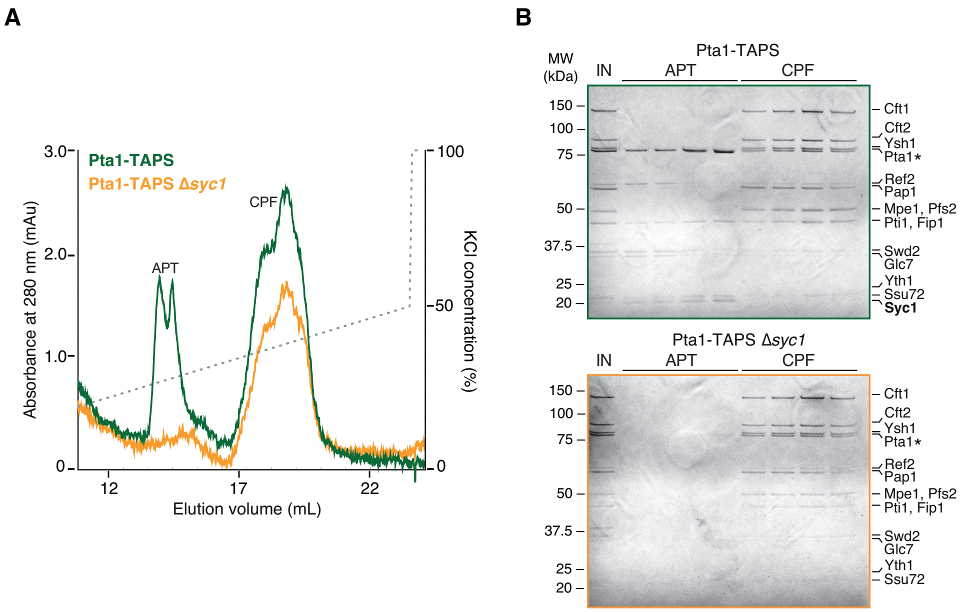

Supplementary Figure S2: Purification from a Pta1-TAPS Δ*syc1* strain does not yield APT. Pta1-TAPS purification from either wild type or Δ*syc1* cells. (**A**) Anion exchange chromatography was monitored through absorbance at 280 nm. The chromatograms from the two runs are overlaid, showing the absence of the APT peak in Pta1-TAPS Δ*syc1* (orange) compared to Pta1-TAPS (green). (**B**) Coomassie-stained SDS-PAGE of the eluted fractions. IN indicates the Streptactin eluate before anion exchange. The subsequent lanes are fractions from anion exchange chromatography with the positions of APT (left) and CPF (right) migration indicated. The upper gel shows that in wild type Pta1-TAPS both APT and CPF complexes are present, whereas only CPF can be detected in Pta1-TAPS Δ*syc1* (bottom gel).

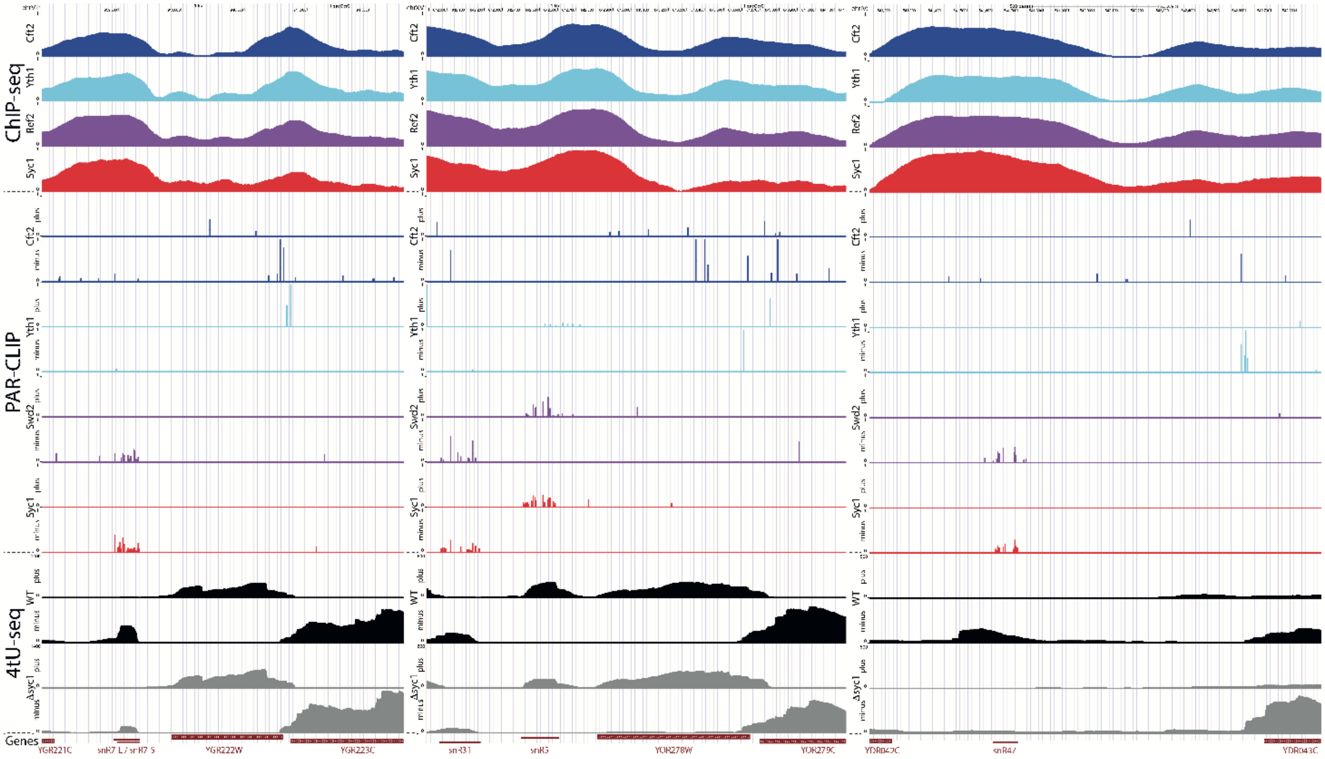

Supplementary Figure S3: Genome-browser tracks. Genome-browser tracks showing normalized ChIP-seq (top), PAR-CLIP (middle) and 4tU-seq (bottom) data at three exemplary genomic regions. Data from replicate measurements have been merged. Ensembl (92) annotations for *S. cerevisiae* (sacCer3) mRNA and sn/snoRNA genes are shown below (top = plus strand, bottom = minus strand).

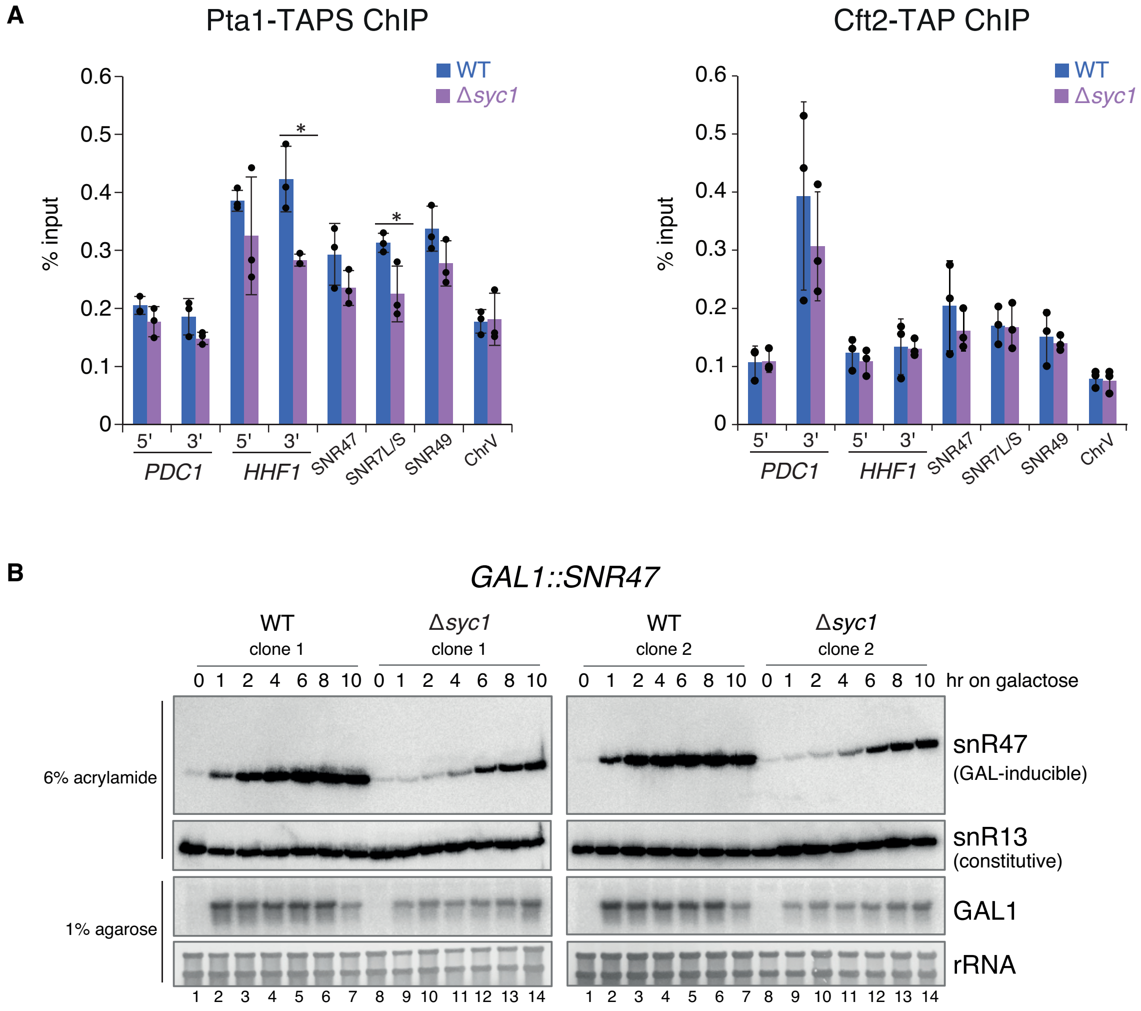

Supplementary Figure S4: Comparison of transcription in wild-type and Δ*syc1* deletion strains. (**A**) Occupancy of Pta1-TAPS (left) and Cft2-TAP (right) from wild-type (WT, blue) and Δ*syc1* (magenta) cells was analyzed by ChIP-qPCR at the indicated genes. *PDC1* is a canonical protein-coding gene; *HHF1* is a histone gene; *SNR47* is a C/D box snoRNA; *SNR7L/S* is two isoforms of U5 snRNA; *SNR49* is a H/ACA box snoRNA; and ChrV is a non-transcribed region. Compared to *PDC1, HHF1* has higher Pta1 occupancy. Transcription of histone genes is repressed by *SYC1* deletion (see Supplementary Figure S5). The average % input (bars) and values for individual replicates (filled circles) are plotted for three independent biological replicates. Error bars represent standard deviation of the mean (*, $p<0.05$, two-sided Student's t-test). (**B**) Northern blots to monitor transcription induction of *SNR47* under the control of the *GAL1* promoter in two independent clones of wild-type and Δ*syc1* yeast strains. snR47 accumulates more slowly in the absence of Syc1 and does not reach wild-type levels even after 10 hours of induction. Wild-type C/D box snoRNA snR13 (not inducible), GAL1 transcript and rRNA were analyzed as controls. *GAL1* induction is also reduced in the Δ*syc1* strain but reaches a maximal level after ~1 hour, unlike snR47. We did not observe any 3′ extended products which would be indicative of a termination defect.

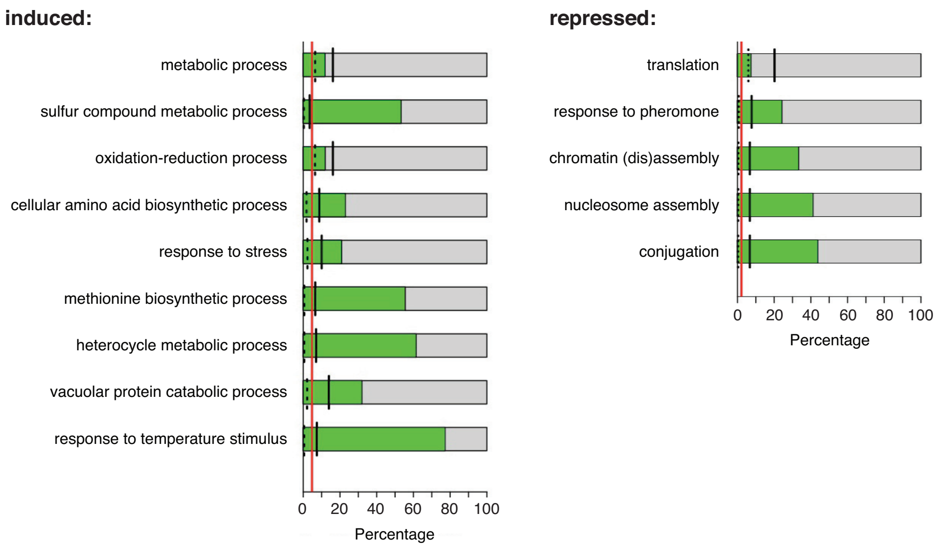

Supplementary Figure S5. Gene Ontology analysis. Gene Ontology (GO) analysis of up-regulated (induced, left) and down-regulated (repressed, right) mRNAs (as shown in Figure 5C). Only 'Biological Process' terms with P value < 10^{-6} and minimum size of 15 genes are displayed, sorted from bottom (most significant) to top. Red line, proportion of induced/repressed transcripts in the whole population. The number of induced/repressed transcripts in the respective GO category is given relative to the GO category size (green bar) and relative to the number of induced/repressed transcripts (black line). Dashed line, relative size of the GO set in the whole population. The GO term 'translation' includes 21 repressed genes, all of which are ribosomal protein genes.

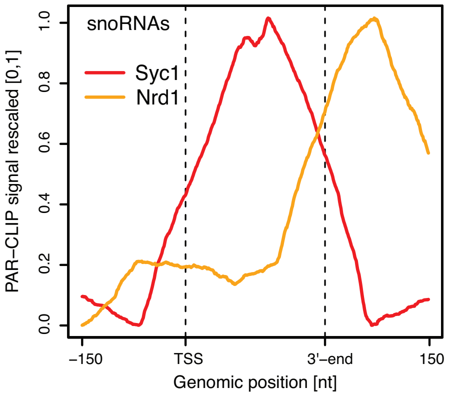

Supplementary Figure S6. Syc1 and Nrd1 have different PAR-CLIP profiles. Gene-averaged PAR-CLIP occupancy profiles over selected sn/snoRNAs for Syc1 (this study) and Nrd1 (52). Before averaging, normalized gene profiles were aligned at their TSS and length-scaled such that their 3'-ends coincided.

Supplementary Table S1. Oligonucleotides

Name	Sequence
PDC1_TSS_F	ACCCAAATCTGATTGCAAGG
PDC1_TSS_R	CAGCTTATGGTGATGGCACA
PDC1_pA_F	AAAGAAGCGGACCCAGACTT
PDC1_pA_R	CCATGGAAAGACCAGACAAGA
HHF1_F	CCAAGCGTCACAGAAAGATTC
HHF1_R	CGCTTGACACCACCTCTTCT
HHF1_pA_F	CATATTTCTCCTAAACCCGCTAT
HHF1_pA_R	AACTGCCCGGTTTTTCTTCT
SNR47_F	TGATGATATCCTATAACAACAACAACA
SNR47_R	TTGTCAAAGTTTGTTTCCACCT
SNR7-LS_F	CGAACATGGTTCTTGCCTTT
SNR7-LS_R	AAAATATGGCAAGCCCACAG
snr49_F	TCTCCATGACTATGCCATTTCT
snr49_R	TCTACGGGATTCGTTTACCA
ChrV-1	GGCTGTCAGA ATATGGGGCCGTAGTA
ChrV-2	CACCCCGAAGCT GCTTTCACAATAC
5GLSNR47	ACCCTAGAAGAAATACCCGAAGATGTAAGGATGTTTTT GTTCCTATAAGCGAATTCGAGCTCGTTTAAA
3GLSNR47 2mce	TTTACATGATTTCAAAAGATATGGAGCTGAAGCCTTTT GTCTTGGCCGAGACTTTTATTACATTTGAATA
Snr47so2	GGACGAAGAAATTCATGTTG
S13so	GGTAGCTTGAGTTTTTCCAC
Gal1PCRsonR	TAAACGGAGTAGCCTTCAAC
